# Realizing the High Q-Factor of a CSIW Microwave Resonator Based on an MDGS for Semisolid Material Characterization

**DOI:** 10.3390/mi14050922

**Published:** 2023-04-24

**Authors:** Ahmed Jamal Abdullah Al-Gburi, Norhanani Abd Rahman, Zahriladha Zakaria, Muhammad Firdaus Akbar

**Affiliations:** 1 Center for Telecommunication Research & Innovation (CeTRI), Faculty of Electrical and Electronic Engineering Technology (FTKEE), Malacca 76100, Malaysia; 2 Centre of Telecommunication Research & Innovation (CeTRI), Fakulti Kejuruteraan Elektronik dan Kejuruteraan Komputer, Universiti Teknikal Malaysia Melaka, Durian Tungal, Melaka 76100, Malaysia; 3 Department of Electrical Engineering, Politeknik Port Dickson (PPD), Port Dickson, Negeri Sembilan 71250, Malaysia; 4 School of Electrical and Electronic Engineering, Universiti Sains Malaysia, Nibong Tebal, Seberang Perai 14300, Malaysia

**Keywords:** circular substrate-integrated waveguide (CSIW), mill-shaped defective ground structure (MDGS), samples under test (SUTs), sensitivity, Q-factor, polypropylene (PP) tube, Javanese turmeric, mango ginger, black turmeric, turmeric, distilled (DI) water, semisolid

## Abstract

In this work, the high-quality factor (Q-factor) and high sensitivity of a circular substrate-integrated waveguide (CSIW) are proposed for the characterization of semisolid materials. The modeled sensor was designed based on the CSIW structure with a mill-shaped defective ground structure (MDGS) to improve measurement sensitivity. The designed sensor oscillates at a single frequency of 2.45 GHz, which was simulated using an Ansys HFSS simulator. Electromagnetic simulation explains the basis of the mode resonance of all two-port resonators. Six variations of the materials under test (SUTs) were simulated and measured, including air (without an SUT), Javanese turmeric, mango ginger, black turmeric, turmeric, and distilled water (DI). A detailed sensitivity calculation was performed for the resonance band at 2.45 GHz. The SUT test mechanism was performed using a polypropylene tube (PP). The samples of dielectric material were filled into the channels of the PP tube and loaded into the center hole of the MDGS. The E-fields around the sensor affect the relationship with the SUTs, resulting in a high Q-factor value. The final sensor had a Q-factor of 700 and a sensitivity of 2.864 at 2.45 GHz. Due to the high sensitivity of the presented sensor for characterization of various semisolid penetrations, the sensor is also of interest for accurate estimation of solute concentration in liquid media. Finally, the relationship between the loss tangent, permittivity, and Q-factor at the resonant frequency were derived and investigated. These results make the presented resonator ideal for the characterization of semisolid materials.

## 1. Introduction

In recent years, there has been a rapid development of interest in microwave resonator sensors for various technological challenges, such as detecting the properties of samples under test (SUTs) and analyzing their structure. Microwave sensors are widely used for material characterization in agriculture, pharmaceuticals, and industry [1,2,3]. Material characterization is necessary when one wants to determine the type of material for a particular application, whether it is a solid, a semisolid, or a powder sample [4,5,6]. Sensor sensitivity is also crucial for microwave engineering when it comes to the properties of characterized materials.

Compared to solid materials, the dielectric constant of liquid materials is more likely to be affected by factors such as temperature, humidity, impurities in the sample holder, atmospheric pressure, etc. [7,8,9]. In addition, experiments with liquid samples are less suitable due to their fluid behavior. Liquid samples consisting of polar particles have a high dielectric constant and loss properties, making the development of in situ dielectric constant and loss tangent experiments for liquid materials a dynamic market [10].

Microwave resonator sensors can be either passive or active devices with a single- or double-port network transmission line that relays the desired electromagnetic wave signal. It has been found that planar microwave sensors with a microstrip split-ring resonator (SRR) and circular substrate-integrated waveguide topologies are capable of producing high Q-factors and sensitivities. Measurement studies have included the dielectric properties of materials and the sensitivity of the resonator based on the perturbation theory. In addition, a wide range of resonators with different specifications has been tested, including resonant frequency, Q-factor, and bandwidth, with an ability to determine material quantities and dielectric characterization of solid, semisolid, and liquid materials in real time. This provides a new, highly suitable method for determining dielectric properties in pharmaceutical, subsurface, chemical, biological, and biomedical applications [11,12,13,14]. Material characterization measurements are conducted by measuring the resonant frequencies of detectors, which can be classified into two types: (1) resonators (sensors) and (2) profound disturbances [15,16]. Compared to broadband techniques, resonance methods can accurately describe material properties at a specific frequency. Microwaves, insulating materials, and coaxial resonators are widely used to transport materials in different environments [17,18,19]. These resonators have been developed to meet industry needs because they are suitable for measuring materials with high accuracy. The resonator can detect various dielectric properties of substrates based on reflection and transmission coefficient characteristics [20,21,22,23,24]. However, achieving good performance with microwave sensors in dielectric and fluid measurements for material detection is still a challenge. Fabrication of these experiments can be complex, and relative permittivity and permeability can be difficult to measure. In this case, planar microwave sensors are preferred due to their many features, such as low cost, simple structures, real-time characterization capabilities, and ease of fabrication [25,26,27,28,29,30]. The integration of cavity sensors lends itself, especially to the substrate-integrated waveguide (SIW) technology, which is inexpensive and promises relatively high-quality and sensitivity values [31]. Many resonators based on SIW cavities have been proposed [32,33,34,35,36]. A standard cavity sensor consists of a cavity resonator printed on a low dielectric loss substrate with a small hole in the center of the cavity and a PP tube inserted through the hole for the samples under test (SUTs) [37,38]. The SUTs provide a deviation of the resonant frequency and the quality factor of the structural cavity mode, which, in turn, allows for the recovery of the dielectric characteristics of the SUT.

Numerous topologies and methods have been proposed in the literature to characterize materials with different liquid concentrations, such as the dielectric constant of liquid samples [39]. One of the most commonly used sensors for liquid detection is the coaxial probe [40,41,42,43], which has the drawback of needing to be immersed in the measured samples. A noninvasive dual-band sensor has been introduced for human health monitoring to detect glucose levels [44] and human milk samples [45]. Two different methods have been presented to characterize the permittivity of the sensor: dumbbell-shaped microwave sensors [46] and slow-wavelength microfluidic integrated waveguides [47].

Complementary split-ring resonators (CSRRs) are considered to be the most typical in the structure of microwave sensors for fluids, and numerous approaches have been used to define the properties of the samples under study [48,49,50,51]. In [48], the sensor was designed and developed in such a way that the liquid remained in its liquid state during the experiments, resulting in a large loss of liquids under test. The microwave sensors proposed by Kiani et al. [49] can effectively evaluate the dielectric constant of liquids, but not the loss tangent of the liquid material. The sensor proposed by Su et al. [50] works with flexible fabrics, which can only be experimented with low-loss materials. At the same time, the sensitivity of the sensor in [51] is too low, at about 150 MHz/mg/mL, and high noise is observed throughout the measurement process.

In this work, the sensor was simulated, tested, and measured using a mixture of Javanese turmeric, mango ginger, black turmeric, turmeric, and distilled water (DI). The rationale for using the substrate-integrated waveguide (SIW) and the structure of the cavity were clearly explained. Adding a mill-shaped defective ground structure (MDGS) in the center of an SIW design improves the distribution of microstrip electric fields, which leads to an increase in the Q-factor [52]. The advantages of the presented sensor using a polypropylene tube (PP) for the SUT channel can prevent sample drop and time corrections [53,54,55]. To test semisolid materials with their high dielectric properties and use in Zingiberaceae families [56], sensors must be highly sensitive to accurately monitor semisolid concentrations. The proposed sensor has a Q-factor of 700 and a sensitivity of 2.864 at 2.45 GHz. Through detailed analysis and experiments, the presented resonator could identify the topologies of the SUTs and detect their concentrations according to the proposed sensors [57,58]. Another important advantage of our proposed sensor is the ease of sample handling and fast measurement repeatability.

## 2. CSIW Sensor Modified with the MDGS Design Configuration

**Figure 1:**
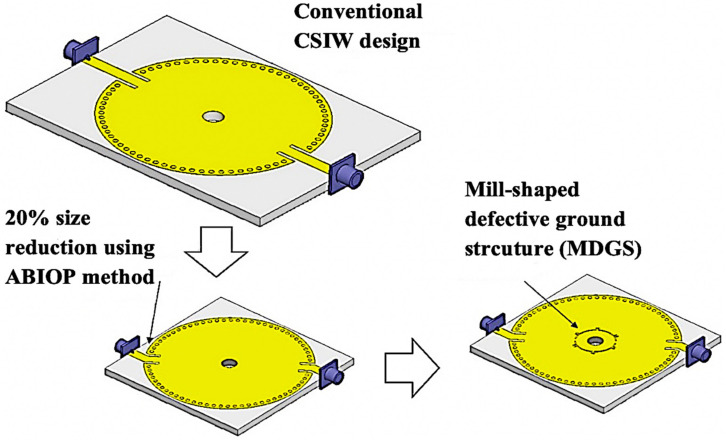
The design process of an MDGS CSIW sensor.

In many RF/microwave applications, the conventional planar resonator sensor utilizing a CSIW structure is commonly utilized. The development of illustrated sensor designs is discussed here through three design processes as demonstrated in Figure 1. The first design was characterized by the conventional CSIW. Next, the angle between the input and output ports (ABIOP) at the CSIW was added to link the two 50-ohm feedlines which regulate the electromagnetic flow for restricting the wave propagating through the device. This has been recently introduced in this CSIW sensor design via an effect on the conventional CSIW of 20% reduction in size. The next step was the introduction of a mill-shaped defective ground structure (MDGS) through the CSIW. The MDGS creates the strongest electric field focus on the center of the CSIW cavity and it is used to control the resonant frequency. It can be noted that to improve the sensitivity and minimize the overall structure, the MDGS CSIW was configured. The MDGS CSIW sensors’ findings were examined and produced outstanding results. Through the use of a lightweight geometric structure, the sensitivity and accuracy of the proposed CSIW sensor were dramatically improved.

### 2.1. Conventional CSIW Sensor

The fundamental analysis of dielectric properties in a micro-volume was defined in this study using a microwave resonator sensor, with emphasis on the CSIW. The proposed sensor operates at a frequency of 2.45 GHz and was developed to characterize the dielectric properties of semisolid materials. RT substrate/duroid 5880 was used for the simulation process due to its low radiation loss and ease of manufacturing with fewer technical processes. The thickness of the substrate (h) was 3.175 mm because a large area inside the substrate needed to be scanned, which is essential for SUT tests. However, a thin copper layer of 0.035 mm is not suitable for testing an SUT on the sensor surface. The details of the physical dimensions of the resonator are given in Table 1.

**Figure 2:**
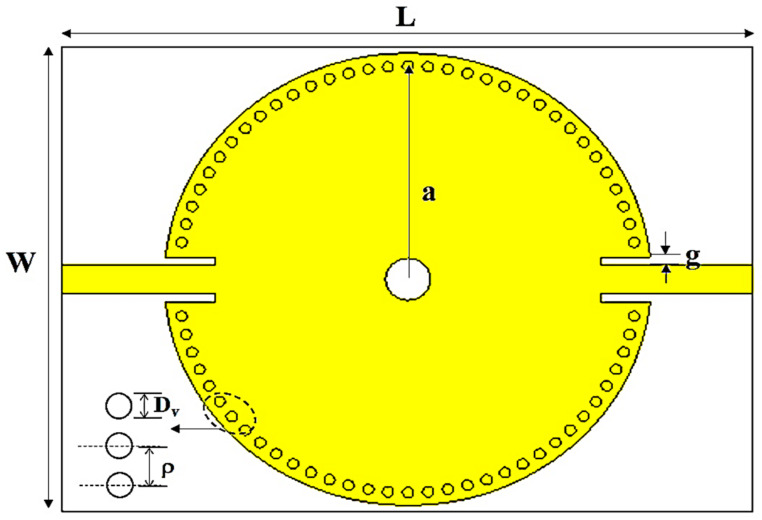
The basic structure of the CSIW resonator sensor.

In this study, some improvements in conventional CSIW sensors were necessary to produce smaller and more sensitive sensors. The method used was to introduce an angle between the input and the output of the feedline port to reduce the size of the structure. Furthermore, to generate a high sensitivity of the sensor, an MDGS structure was introduced to obtain the strongest E-fields in the sensing area. This type of conventional CSIW resonator sensors has an overall dimension of 86.3 mm × 69 mm × 3.245 mm (L × W × h). The physical layout of the circular substrate-integrated waveguide is shown in Figure 2, where every view is shown in detail. The material used is the same as for the previous design, which is RT/duroid 5880, but with a different copper plating.

A calculation was conducted to identify the sensor sensitivity to changes in the permittivity of the surrounding material. The radius of the conventional CSIW sensor based on Equation (1) was calculated as follows:

(1) $$a=\frac{T_{m,n}\times \;C}{2\pi f_{r}\sqrt{\varepsilon_{r}}}=\frac{2.405\times 3\times 10^{8}}{2\pi \left(2.45\times 10^{9}\right)(\sqrt{2.2)}}=31.6\;\mathrm{mm}\;$$

The value of *T_m_*_,*n*_ for the Bessel function values used for measuring the circular waveguide distance is shown in Table 2, where *C* denotes the speed of light, $\varepsilon_{r}$ is the dielectric constant of the substrate, and $f_{r}$ is the center frequency. Using the design rules, the hole diameter (*D_V_*) of the CSIW and its pitch (*ρ*) can be calculated through Equations (2) and (3), respectively.

*D_V_* > 0.2 *λ_o_* (2)

The distance between the holes can be obtained using Equation (3):

(3) $$\frac{D_{V}\;}{\rho }\leq 0.5\;$$

### 2.2. Angle between the Input and Output Ports (ABIOP)

**Figure 3:**
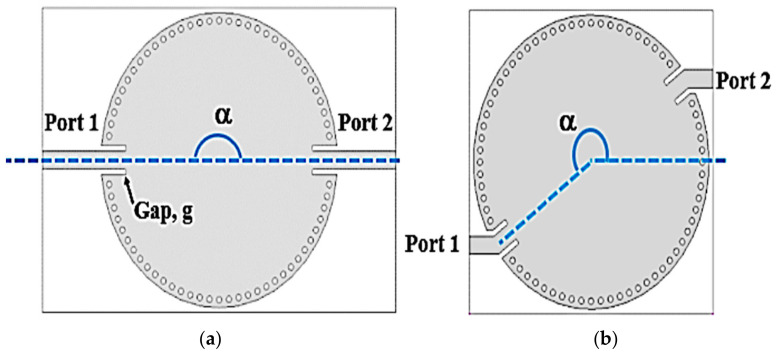
Configuration of the CSIW model. (**a**) Conventional CSIW with ABIOP 180°. (**b**) Proposed new CSIW with an α ABIOP.

Typically, in traditional CSIW cavity sensor designs, the input and output ports are placed in line at 180 degrees, resulting in a sharp roll-off characteristic in the passband frequency response of S21. However, this sensor design prototype is often quite large, particularly at lower resonant frequencies. To address this issue, the researchers proposed a new method utilizing a bend-coupled microstrip with 45-degree angle bends referred to as the ABIOP which is inserted into the transmission line between the input and output ports. The main goal of this approach is to reduce the size of the sensor while increasing its Q-factor. Consequently, they investigated the ABIOP technique with two 50-ohm feedlines regulating the electromagnetic flow, restricting the transmission of waves. By identifying the optimal angle for the ABIOP, they introduce a new CSIW sensor design that reduces size by 20% compared to the conventional design, as shown in Figure 3.

**Figure 4:**
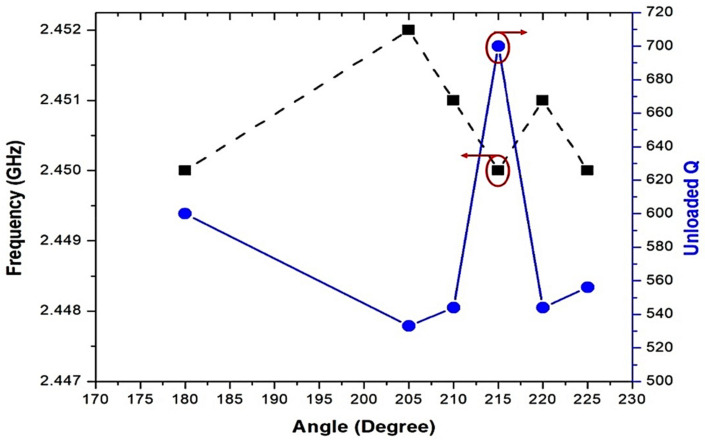
Simulated frequency and unloaded quality factor of the CSIW designed using the ABIOP technique.

The high-frequency structure simulator (HFSS) shown in Figure 4 was used to simulate different ABIOPs. When α ABIOP increased, the unloaded Q-factor generated as per Equation (4) also increased and could exceed 700 for the best optimal angle. This means that a lower Q-factor element is associated with angles greater than α = 215°. To monitor the effect of sensor capacitance, the coupling gaps of the ABIOP were set at g = 1.2 mm, and 66 plated hole vias were used in a circular form grounded to the ground using the top circular patch as a waveguide excitation. The optimal angle α between the input and output ports of a circular SIW cavity was experimentally and theoretically determined to be 75°, which differs from the generally accepted 180° (in-line). By connecting circular SIW cavities with the optimal angle α, a high-output millimeter-wave SIW sensor can be created. The unloaded Q-factor (*Q_u_*) can be determined as defined in Equation (4) [52]:

(4) $$Q_{u}=\frac{2f_{0}}{\Delta f}\;$$

Meanwhile, the value of Δ*f* is set at −3 dB for the bandwidth, considering the lowest frequency shifts.

**Figure 5:**
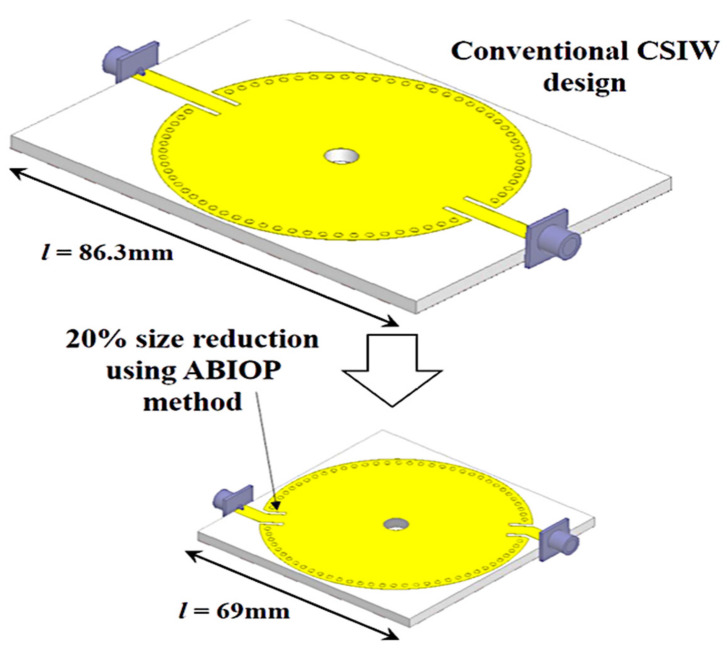
A new structure was created by reducing the size of the conventional CSIW substrate by 20%.

Conventional CSIW devices are limited by their large size, particularly at lower frequencies. To address this issue and reduce their size, a technique called the angle between the input and output ports (ABIOP) was employed in the CSIW. This technique allows the two 50 Ω feedlines that control the electromagnetic flow and restrict wave propagation in the system to be connected. Recently, a new CSIW sensor design has been developed that reduces the size of the conventional CSIW substrate by 20%, as illustrated in Figure 5. The figure shows that the length of the original substrate has been decreased from 86.3 mm to 69 mm.

### 2.3. Determination of Modes and Resonant Frequency (ABIOP Design)

**Figure 6:**
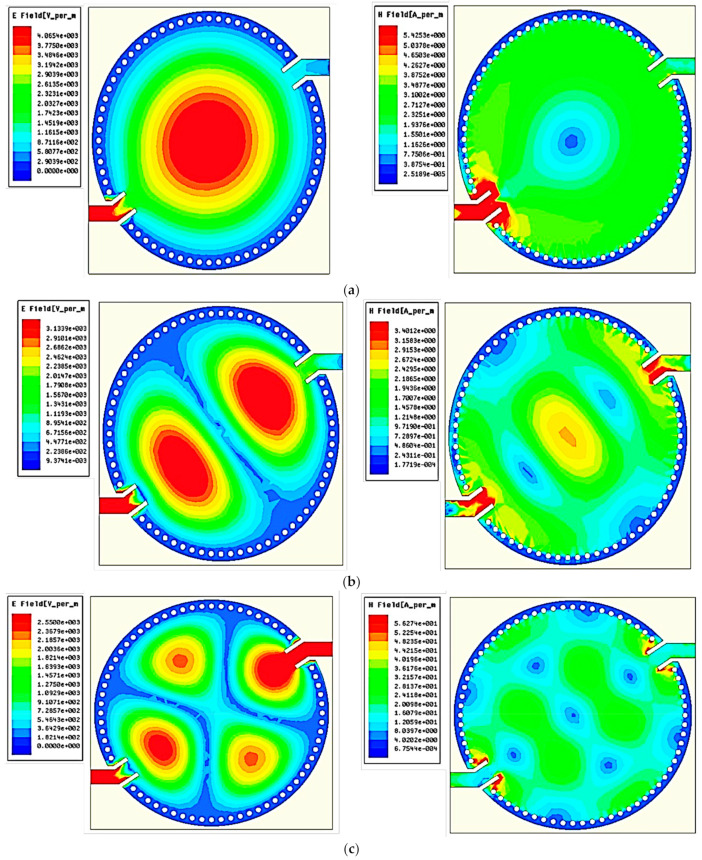

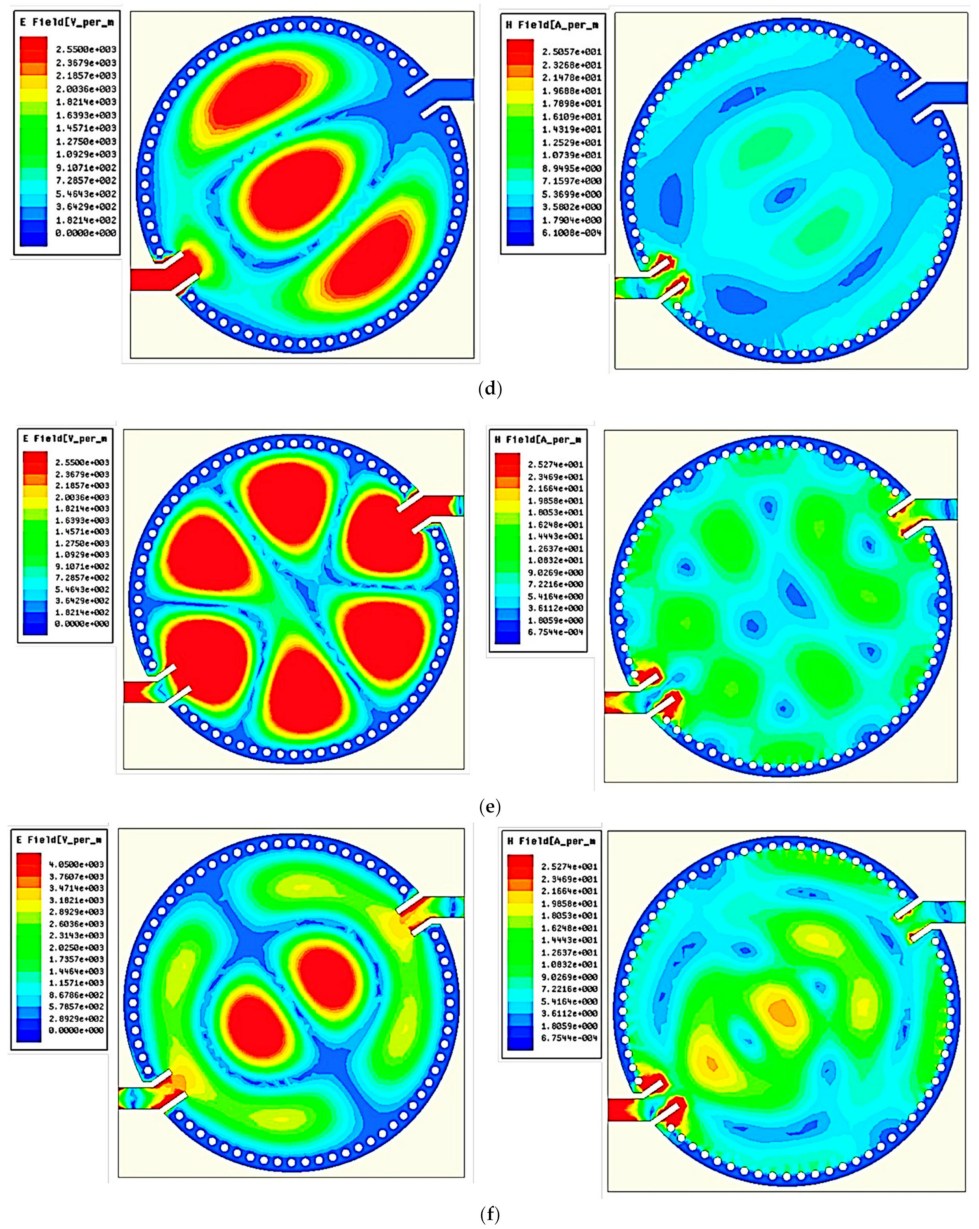
E- and H-fields of an unloaded cavity for (**a**) TM_010_, (**b**) TM_110_, (**c**) TM_210_, (**d**) TM_020_, (**e**) TM_310_, and (**f**) TM_120_.

An EM simulator was used to ensure the CSIW calculation and simulation resonant frequencies were similar as shown in Figure 6. A CSIW resonator was simulated to generate the numerous modes, and it propagated E-fields/H-fields of TM modes at different frequencies. In the substrate cavity, there is an unlimited number of modes. Some lowest modes were selected for research purposes, as shown in Table 3, through the 31.6 mm radius value extracted from Equation (1) with the substrate’s dielectric constant *ε_r_* = 2.2.

Based on the mode study conducted, the design technique mapped the circular SIW’s cutoff frequency for the TM_010_ mode. As a consequence, the high-concentration flux density at the center of the substrate should give the sensor a high performance. The selection of this mode is also dependent on the use of the sample holder integrated into the middle of the substrate.

### 2.4. MDGS CSIW Design Structure

**Figure 7:**
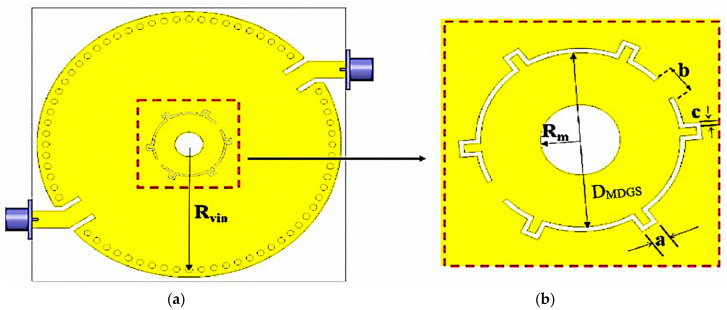
CSIW resonator design structure. (**a**) Top view of the MDGS CSIW. (**b**) Focusing on the MDGS structure parameters.

**Figure 8:**
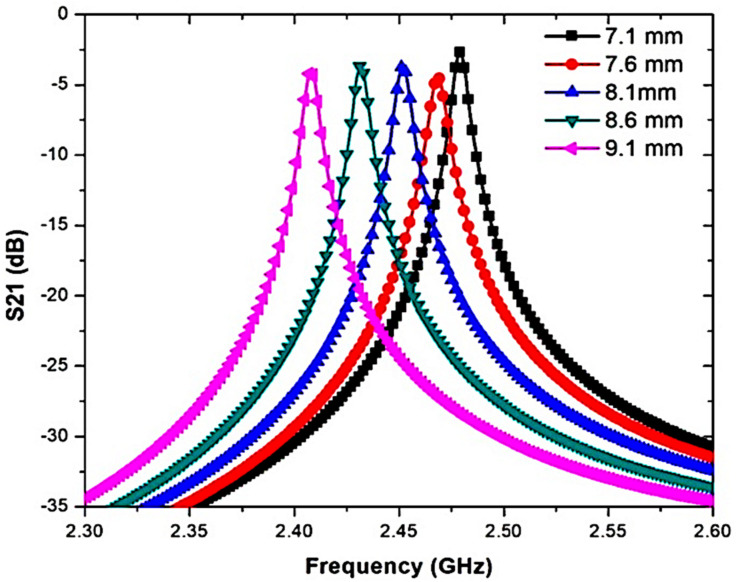
Simulation results for several diameters of the MDGS.

In this section, electric field distribution in the presence of a mill-shaped DGS inscription on the CSIW surface was discussed. The findings indicate that the radius of the circle is a crucial element to control the resonant frequency and produce a high Q-factor. It is possible to obtain the right resonant frequency at 2.45 GHz by arranging the number of diameters of the DGS circle. Therefore, a DGS in a square shape array, namely mill shapes, was created to have better accuracy of the sensor. The original concept of an MDGS is to develop the length of the shape of a DGS that is longer than the initial circumference of the circle and to give maximum limits of electric fields in the central area of the substrate. This effect helps to control the resonant frequency to the desired value and even increases the Q-factor value. The MDGS is also utilized to focus back on field distribution. Overall, the resonant cavity does not have as much energy dissipation in the standard form, thus making the effective capacitance and inductance increase. A parametric study of DGSs focuses on the radius of the MDGS, square array sizes, and angle between two MDGSs. The effect of the radius on the maximum value of the electric field is presented in Figure 7 and Figure 8 and tabulated in Table 4.

The enhanced sensitivity of the CSIW sensor was translated as the starting of the CSRR structure potentially increases the quantity of adsorbed water molecules on the substrate surface, thereby increasing the effective dielectric constant. As a consequence, the shunt LC resonant tank’s equal capacitance increases. Other than that, the fringe electric fields of the CSIW sensor may even raise the equivalent capacitance of the LC resonant tank. Both variables would greatly lower the resonant frequency of the CSRR-loaded CSIW sensor [60].

**Figure 9:**
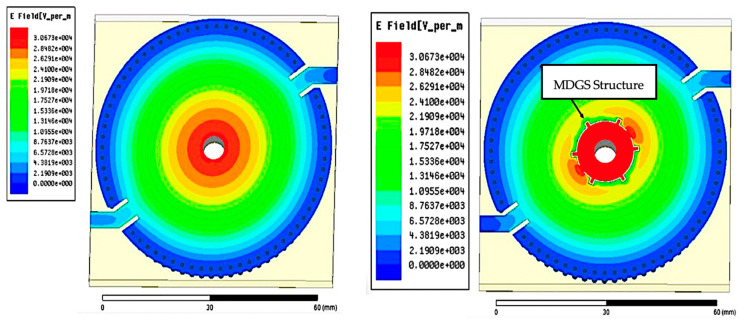
The differences in electric field distribution of the TM_01_ mode when the MDGS configuration was added to the suggested sensor.

The stronger electric field can generate focus around the 6.2 mm diameter hole. As shown in Figure 9, a weak electric field is observed around the hole. Meanwhile, after the implementation of a MDGS, the improvement of electric fields up to 3.0673 × 104 v/m was as shown in Figure 9, with a low return loss of 3.561 dB. It proves that the proposed sensor has a stronger electric field in the SIW cavity with a high Q-factor compared with other resonator sensors at 2.45 GHz [61,62].

**Figure 10:**
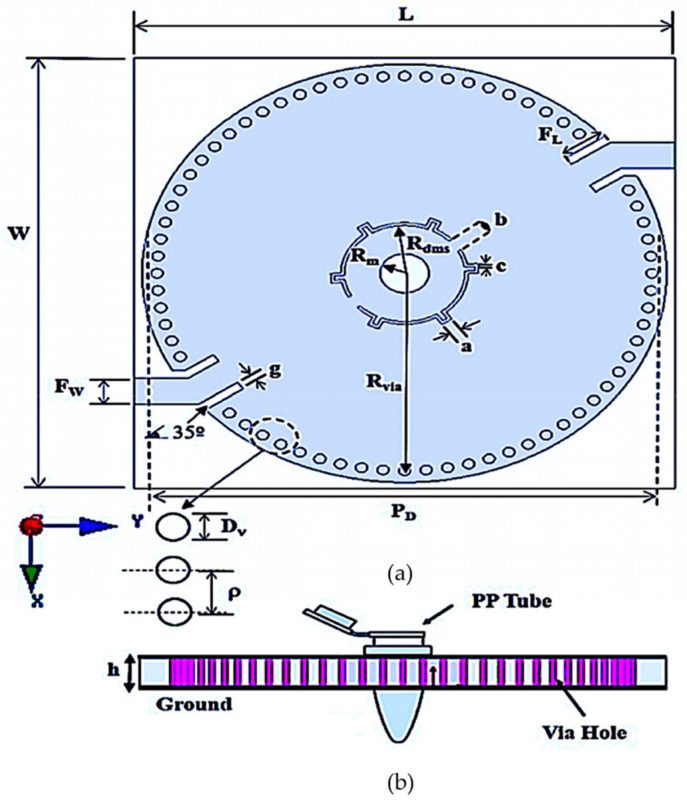
Geometry of an MGDS CSIW sensor. (**a**) Top perspective. (**b**) Sensor illustration with a PP tube. L = 69, W = 69, h = 3.175, t = 0.035, R_m_ = 6.2, R_dms_ = 4.05, F_w_ = 4.2, R_via_ = 31.6, *D_v_* = 1.5, *ρ* = 2.8, d = 1.5, F_L_ = 4.78, R_h_ = 3.1, b = 2, a = 1.5, c = 0.4, g = 1.2. All dimensions are in millimeters (mm).

Simulation specifications are executed by an HFSS to analyze the electromagnetic wave propagation of the proposed topology towards the sensor prototype. The design specifications and geometric parameters of the proposed MDGS CSIW structure are showed in Figure 10.

The physical layout of an MDGS CSIW structure resonator is presented in Figure 10 where each physical dimension is shown in detail to rework as required by any desired frequency. The selection of substrate material is identical to an earlier version that is RT/duroid 5880 laminated with electrodeposited copper of 1 ounce (35 μm) on both sides and masked with gold conductivity to protect the copper plate layers.

## 3. Samples under Test (SUTs)

The channel slot operates a PP tube loaded in the middle of the CSIW cavity, which is the most sensitive region of the dielectric substrate. It is the best choice because it allows the highest electric field arrows to pass through the sample medium in the tube, making the interaction with the electric field strands more robust overall. A SUT is inserted in the polypropylene (PP) tube with a 2.1 dielectric constant value of the polypropylene material.

**Figure 11:**
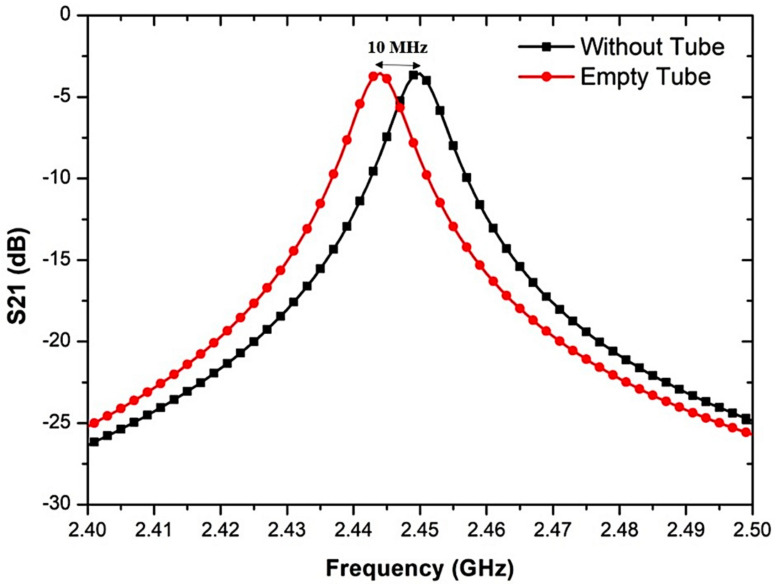
Effect of the frequency shift when the PP tube is loaded into the MDGS CSIW sensor.

A small change in the resonant frequency as revealed in Figure 11 with 10 MHz occurs when the PP tube is placed into the cavity. The electrical field of the slot interacts with the PP tube and energy is related to it and allows the resonant frequency to shift. This shift depends on the permittivity and is independent of the dielectric loss of the tube.

**Figure 12:**
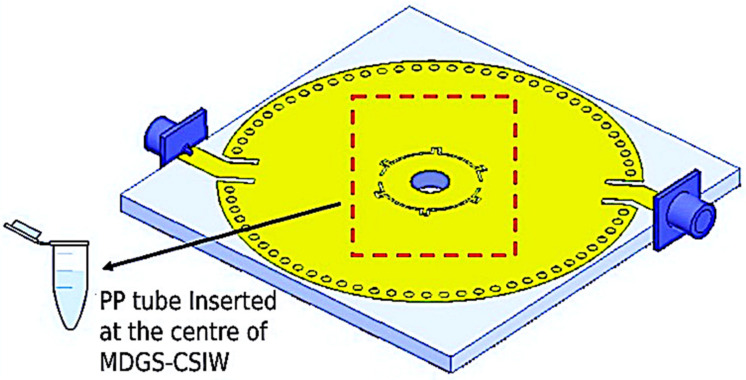
Graphical view of an MDGS CSIW resonator integrated with the PP tube channel.

The sampling principle aims to catch differences in the resonant frequency and insertion loss of the channel slot. From this, an interaction between the radiated near field and the encapsulated solvent in the channel can be inferred. The maximum electric fields provide information about the cavity of the channel slot of the device. The close-up view is shown in Figure 12 to deliver an indication of the detection area.

**Figure 13:**
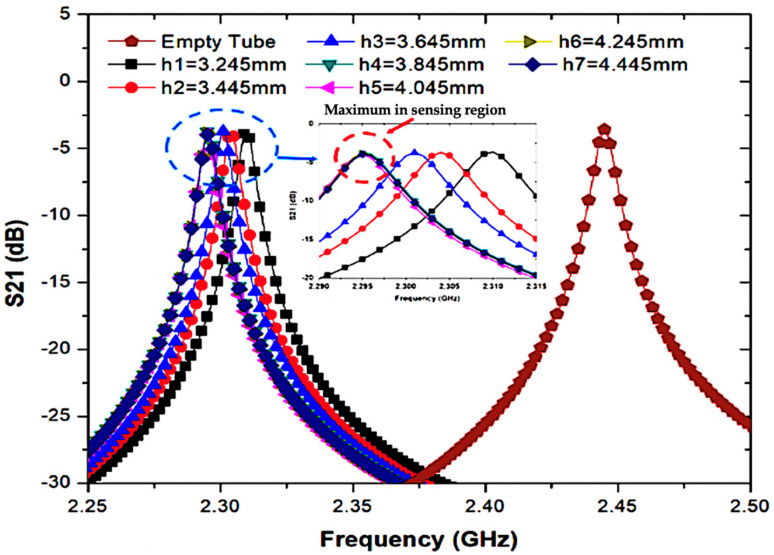
Simulated frequency shift at different volume levels of the sensing area using distilled water in the PP tube.

Therefore, based on the tube volume length, the most promising execution can be performed when the highest frequency change arrives at the saturation level (h) while the liquid volumes are consumed. In Figure 13, the simulated S21 of the suggested sensor with an empty 6 mm tube filled with distilled water indicates that the most suitable volume length is 3.845 mm, which corresponds to 0.11 mL of a semisolid material. A PP tube with a low dielectric constant *ε*′ of 2.2 was selected because the lowest frequency shifted when presenting the tube, so that sample handling is easier, without contamination during operation, and as repetition measurements can be conducted fast. Meanwhile, the effect on the field propagation of the CSIW can also be bypassed based on the specification of the wavelength over *λ*g/20, which is larger than the diameter of the tube.

**Figure 14:**
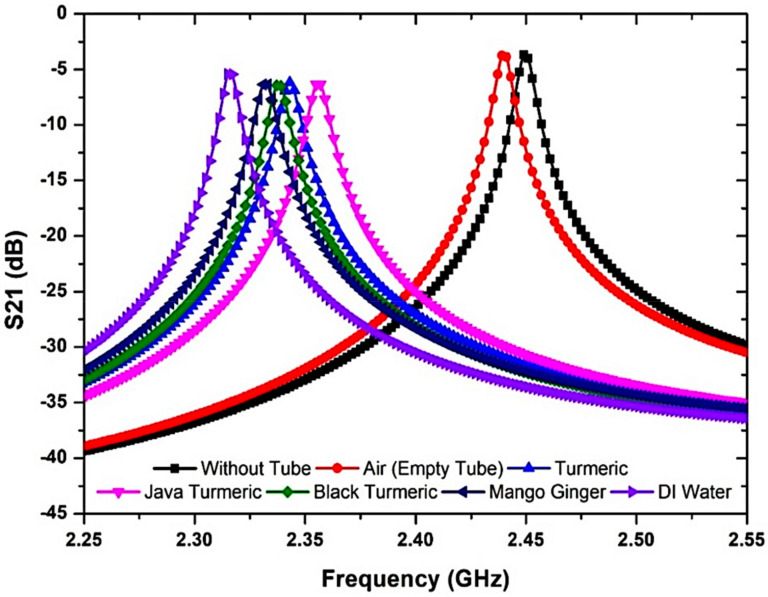
Frequency response of the MDGS CSIW sensor with the presence of semisolid materials.

To prove the sensor achievement, the sensor that was suggested was evaluated using several semisolid materials as SUTs. Furthermore, by using the permittivity value provided by Aziz et al. [56], the semisolid samples of Javanese turmeric, mango ginger, black turmeric, and turmeric had an *ε*′ of 34.52, 45.6, 46.68, and 58.61, respectively, at less than the 2.5 GHz resonant frequency. The frequency response of the MDGS CSIW sensor was demonstrated with several SUTs with different dielectric properties as illustrated in Figure 14. Table 5 shows the outcomes of the frequency reaction studies when the SUTs were used.

Consistent with the findings of Jha and Akhtar [61], the minor resonant frequency shift was observed to be proportional to the dielectric constant. Nevertheless, in this simulation, distilled water perturbed more electric fields into the cavity. A higher dielectric constant of the SUTs perturbed the electric flux more, which shifted to low frequency. The findings revealed a good correlation between the simulation and the theoretical conception.

## 4. Fabrication and Measurement

**Figure 15:**
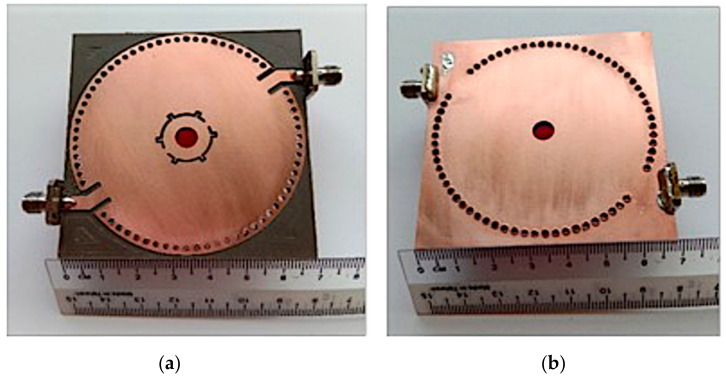
Fabricated MDGS CSIW sensor. (**a**) Top view. (**b**) Bottom view.

In this study, the fabrication and sample preparation for measurement is evidence of this thesis objective for these research works. Several types of test samples, such as liquid and semisolid samples, were prepared for the validation of sensors in this work. The MDGS CSIW sensor was printed on a Roger RT/duriod 5880 substrate with relative permittivity *ε_r_* = 2.2, loss tangent of 0.0009, thickness of the substrate and the conductor of 3.175 mm and 0.07 mm, respectively. The fabricated prototype of the proposed MDGS CSIW resonator is shown in Figure 15a,b. The vias of the circular waveguide were drilled with a Burgard CNC machine with a diameter of 0.75 mm. Then, the nonconducting vias of the circular waveguide metallic wall substrate coated with a thin layer of chemical copper were electroplated using the plated through-hole (PTH) process, whereas the top patch layers were fabricated using the standard photolithography technique and the PCB etching process to defect the patch for the teeth gear shape. The final step was to drill a 6.2 mm diameter hole on the substratum for the tube channel by the CNC machine.

**Figure 16:**
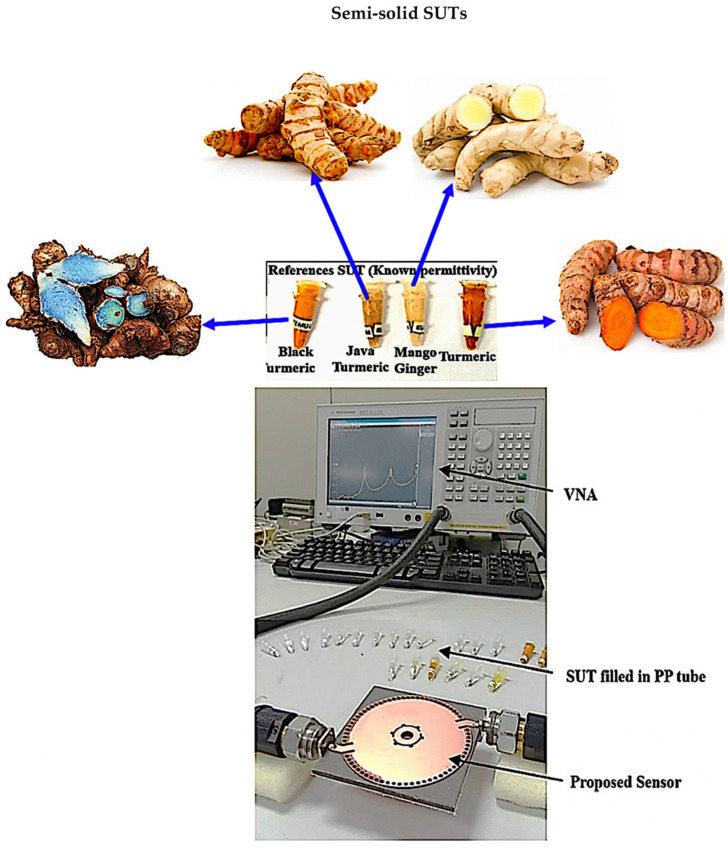
Experimental setup for the MDGS CSIW sensor.

The S21 properties of the manufactured MDGS CSIW sensor were measured with a range of 1 to 5 GHz using an Agilent E5071C network analyzer and photographs of the experimental setup along with the semisolid Zingiberaceacae samples are shown in Figure 16.

**Figure 17:**
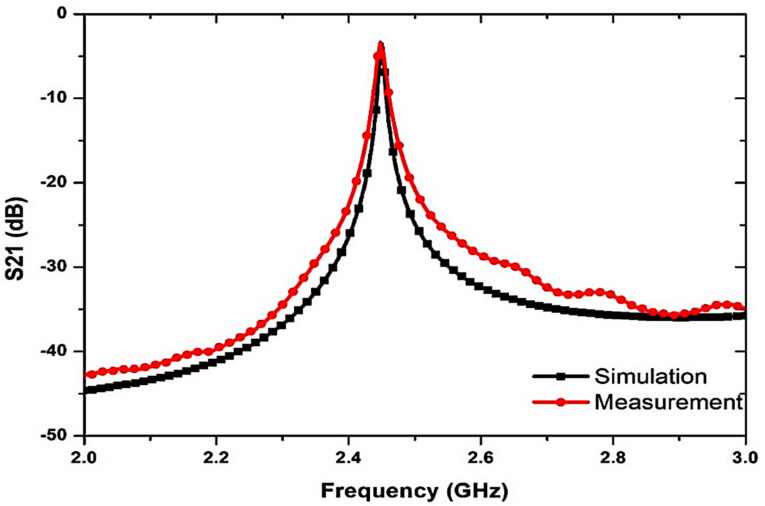
Simulated and measured results of the MDGS CSIW sensor.

Figure 17 shows the comparison between the simulated and measured responses. The sensor response was estimated and documented during the experimentation when the SUTs were loaded. The PP tube was placed in the center of the MDGS CSIW sensor in order to evaluate the dielectric materials of the semisolid samples.

**Figure 18:**
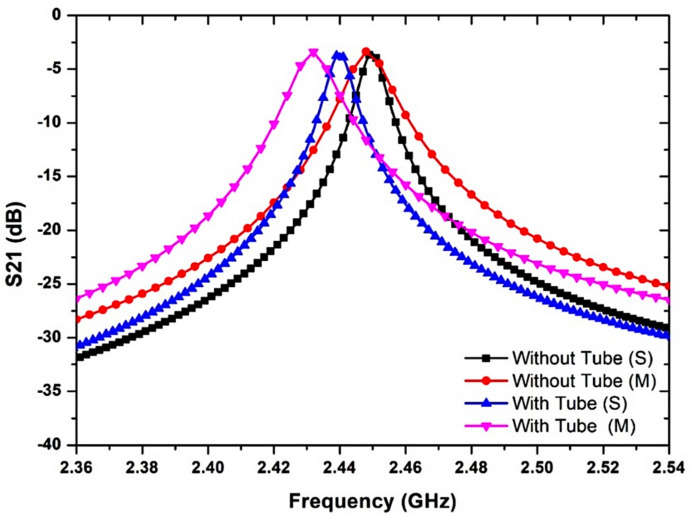
Simulated and measured results of the MDGS CSIW sensor with S-parameter output with and without a tube.

The MDGS CSIW sensor and the S-parameter for the comparison between the simulated and measured responses were provided in Figure 18 when filling the PP tube. The findings suggest that the proposed sensor exhibits bandpass transmitting features where the resonant frequency is dependent on the size and configuration of the structure of the microstrip. According to Figure 18, the resonant frequency experienced a slight alteration of 10 MHz when the PP tube was inserted into the cavity. This change occurred because the electrical field of the slot interacted with the PP tube and transferred energy to it, resulting in a shift in the resonant frequency. The degree of this shift was determined by the permittivity of the tube and was not affected by its dielectric loss.

Table 6 shows that the frequency of the resonance measurement of the fabricated structure is 2.448 GHz compared to 2.45 GHz in the simulation. The difference between the simulation and the measured result was mainly due to the inconsistency between the SMA connectors and the feedlines and to the manufacturing tolerance and simulation accuracy limits.

## 5. Data Analysis

In order to achieve the optimum performance, the results of the simulation were evaluated using synthesis studies and optimization. In this study, the simulation review and measurement results were compared and the SUT data repetition process was performed. At the same time, complex permittivity values, sensitivity, and ethanol concentrations in SUT liquids were also analyzed. Several semisolid SUTs were measured to validate the sensor efficiency from 1 to 5 GHz using VNA. To minimize the liquid waste of the samples and time measurement, the polypropylene (PP) tube with a lid was used as a liquid channel. The minimum amount of liquid of 0.11 mL was filled in the tube, covering the sensing area. Besides, sample handling was simple, and the measurement could be repeated quickly. Moreover, by contrasting the measured data between the sensor and the current commercial sensor, the validity of the data results was verified. A constant temperature room was also standardized, and the measurements were repeated three times to produce an accurate result with respect to the average data values. The change in the frequency response was evaluated and found to be comparable with the simulated results in order to ensure the same performance. The polynomial fitting technique was implemented, and from these datasets, a numerical expression was created. The working principle contributed to the identification of the complex permittivity, loss tangent, concentration, and sensitivity of the proposed sensor.

### 5.1. Repeatability of the SUTs

**Figure 19:**
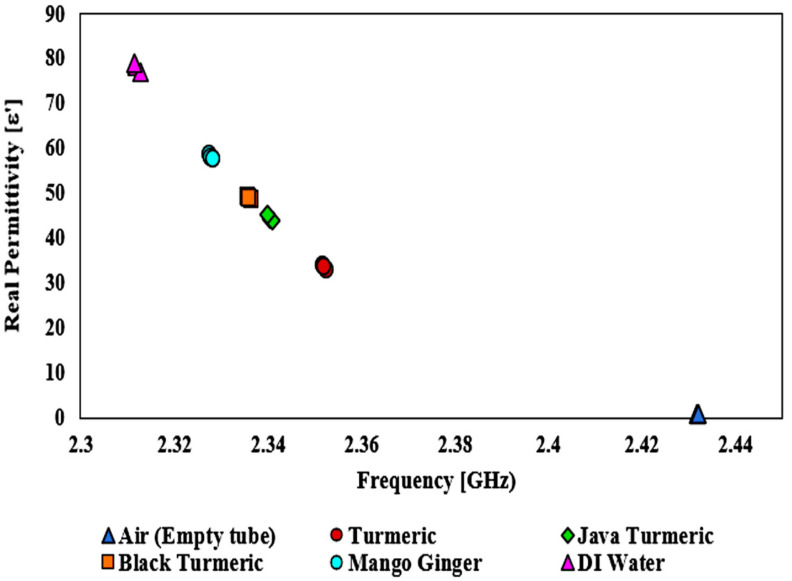
Repeated measurements of the MDGS CSIW sensor for semisolid samples.

Figure 19 and Table 7 indicate the presented sensor estimated the dielectric value of the different SUTs. The measurement would be completed in three minutes for a threefold measurement in one cycle to ensure short-term repeatability. The replicated measurements deliver an actual result of the average data values ($\tilde{x}$) and the interpretations of the minimal outcomes available. In most sensor measurements, the effects of the collected data demonstrated good repeatability. The use of PP tubes such as SUT channels was also a noncontact sample for liquid and semisolid materials, which simplified and accelerated the repeat cycle.

### 5.2. Semisolid SUTs

**Figure 20:**
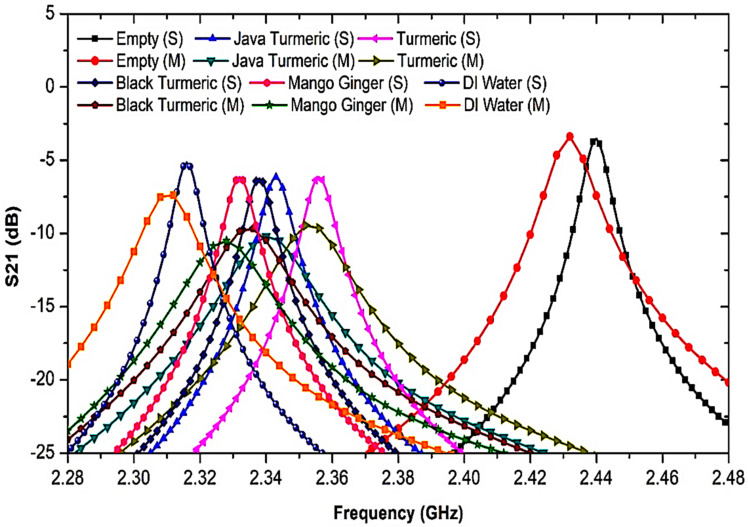
Simulated and measured S21 responses with several permittivities of semisolid SUTs.

Different analyses of dielectric properties were conducted with Javanese turmeric, mango ginger, black turmeric, and turmeric for the reliability and validation of the proposed sensor using semisolid SUTs. Figure 20 shows the S-parameter of the frequency response with a different permittivity of SUTs at 2.45 GHz. As a result, a significant trend of the declining peak amplitude of simulation outcomes led to the decreasing sensitivity of the sensor. The shift in the resonant frequency is known as the information pertaining to the permittivity (*ε_r_*) of a SUT.

As can be seen, the resonant frequency shifted to a lower frequency when the permittivity value of the sample was increased. This is because the effective inductance and capacitance parameters of the circuit became greater than those of the SUT. It was found that the higher the dielectric constant of the SUT, the lower the resonant frequency [63]. In the perturbation technique, the movement of the resonant frequency depends on the response between the dielectric materials and the E-field distribution of the resonator. This exchange makes perturbation toward the electrons of the SUT in the electric flux density generating a difference in the frequency response and affects the Q-factor magnitude. The semisolid SUTs were investigated when positioned in the middle of the MDGS CSIW sensor which demonstrated an interaction between the electric field and SUTs led to a change in the resonant frequency. Table 8 displays the proposed sensor’s simulated S-parameter data after loading an SUT channel.

**Figure 21:**
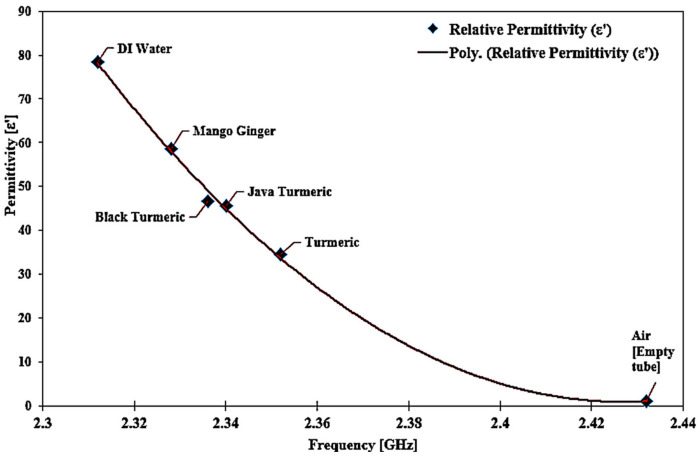
Relationship of the known permittivity to extract unknown material properties (semisolid samples).

Dielectric properties are known to be sensitive and quickly influenced by temperature variation. Therefore, keeping a constant temperature in the room and performing regular calculations would standardize the process and provide reliable outcomes for the average data values. The technique of polynomial fitting was utilized to define the permittivity of the unknown SUTs. This method can also be extended to the sensing medium with careful attention to the sample properties, frequency response, and thorough investigations. The equation could potentially differentiate between two separate forms of solutions. Figure 21 shows that the contrast between the two datasets (reference and simulated) was evaluated with the help of the SUTs dependent on the permittivity. To measure the permittivity of the standard samples, the frequency shift related to each sample could be utilized as well.

The curve-fitting method (second-order polynomial) that can be applied to obtain this expression is as follows (5):

*ε*′ = 5879.2*f*^2^ − 28,533*f* + 34,620 (5)

**Figure 22:**
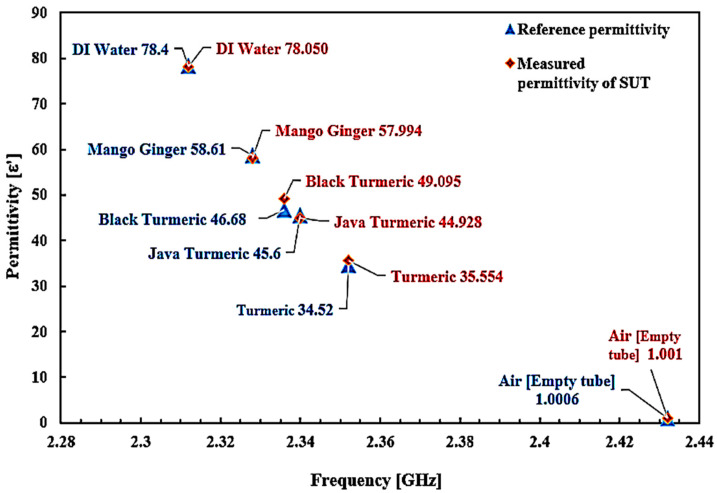
Comparison of the ideal and measured real-part permittivity of several semisolid samples.

At this stage, it is possible to derive the dielectric constant of any semisolid sample (unknown permittivity) from a particular equation. The percentage error function and the standard error trendline of the dielectric constant can be seen in Figure 22.

Table 9 summarizes the measured permittivity values. A particular substance has specific permittivity values. The frequency shift represents the component properties themselves. For other terms, permittivity may be derived from the response to the frequency shift. Hence, dependent on that essential permittivity parameter, one can accurately calculate the quality and safety of the components.

Sensor execution is specified by measuring the permittivity (dielectric constant) of SUTs based on the change in the resonant frequency. The outcome indicated satisfactory achievement with different dielectric values as shown in Figure 20. Each single substance has specific values of permittivity. Frequency shifts represent the properties of the substance itself. This explicitly indicates that the resonant frequency shifted down due to the increasing value of *ε*′ of the SUT. For example, in the empty case with the *ε*′ of 1, the resonant frequency was 2.432 GHz, and for the turmeric case with the *ε*′ of 34.52, the resonant frequency decreased by 96 MHz, for Javanese turmeric and black turmeric—by 106 and 112 MHz with the permittivity of 45.6 and 46.68, respectively. It can be seen that the MDGS CSIW sensor can identify and characterize materials with a small variation in material properties.

There is very good agreement when this is compared with other studies in terms of the dielectric constant [56]. This proposed approach has a tolerance average of ±1.86% error detection of the MDGS CSIW sensor, with the minimum and maximum errors of 0.28% and 10.03%, respectively. Compared with the commercial sensor, the tolerance average of ±18.2% error detection was recorded. These parameters were challenging to measure accurately because of several practical difficulties in the manufacturing process, which are slightly different dimensional parameters from the simulation model. Here, we can see further comprehensive improvements made to the sensitivity of the MDGS CSIW sensor to characterize materials with regard to a planar framework.

**Figure 23:**
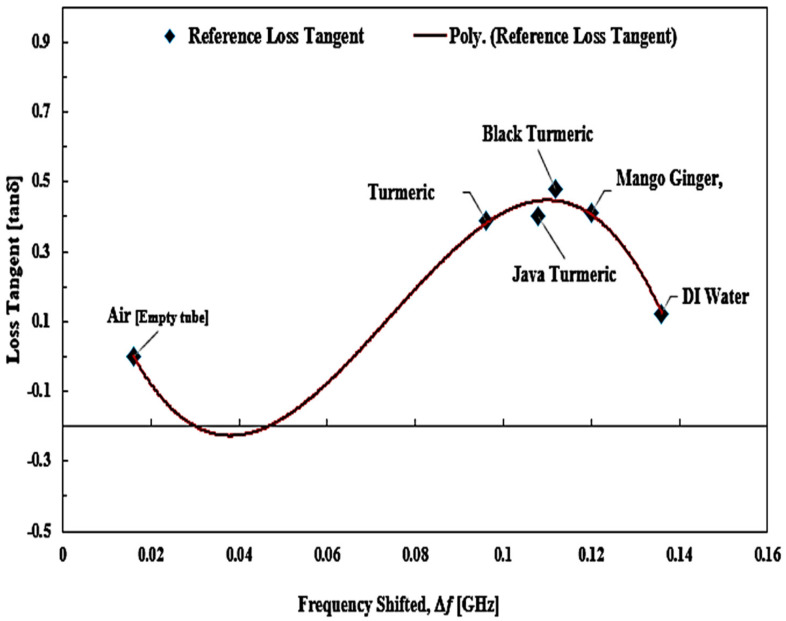
The polynomial fit of semisolid SUTs, loss tangent.

**Figure 24:**
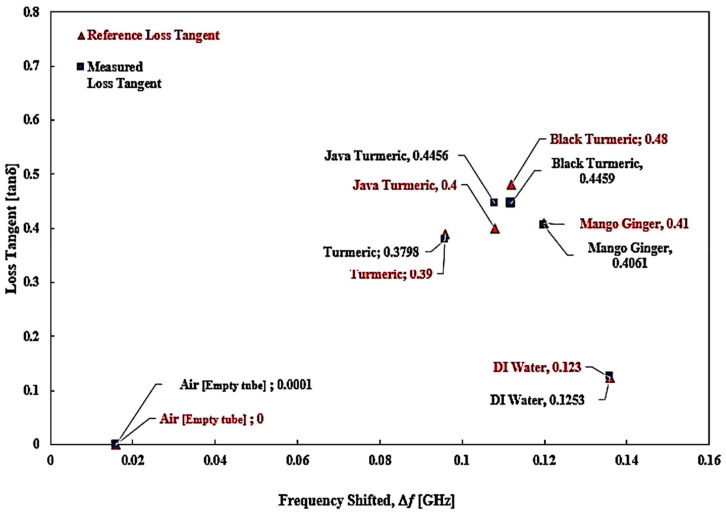
The ideal and measured loss tangent of several semisolid SUTs.

A mathematical model of the curve-fitting technique for the determination of loss tangent (*tan δ*) and imaginary portion (*ε*″) of the complex permittivity was used to monitor and analyze the frequency shift (Δ*f*) of the SUTs. A graphical description of the relationship between loss tangent and resonant frequency shift from that specific data collection with regard to the ideal loss tangent is presented in Figure 23, while the trendlines for the loss tangent percent error between the reference and the measured ones are highlighted in Figure 24.

Reference loss tangent data for the SUTs are denoted by red triangular points and the measured data are shown as blue square shapes with a blue line polynomial fit of loss tangent. It can be found that there is a *tan δ* distribution. The Δ*f* was not constant, and the distribution is provided by Equation (6). However, the relationship between the two parameters can be expressed as a third-order polynomial term to generate an efficient number equation as defined instead of as a second-order relationship.

*tan δ* = −3590.8(|Δ*f*|)^3^ + 797.96(|Δ*f*|)^2^ − 45.098(|Δ*f*|) + 0.5321 (6)

The results of this analysis are summarized in Table 10. Based on the available data, it can be proposed that the MDGS CSIW sensor provided a good minimum tolerance of measurement errors with a value of ±4% compared with that of the commercial sensor, ±28.3%.

Both the reference method and the proposed method showed a very similar performance in the loss tangent values. The difference for Javanese turmeric was 11.4%, 7.1% for black turmeric, 2.6% for turmeric, and 1% for mango ginger. The air loss tangent assumes zero due to the standard dielectric properties of the material.

**Figure 25:**
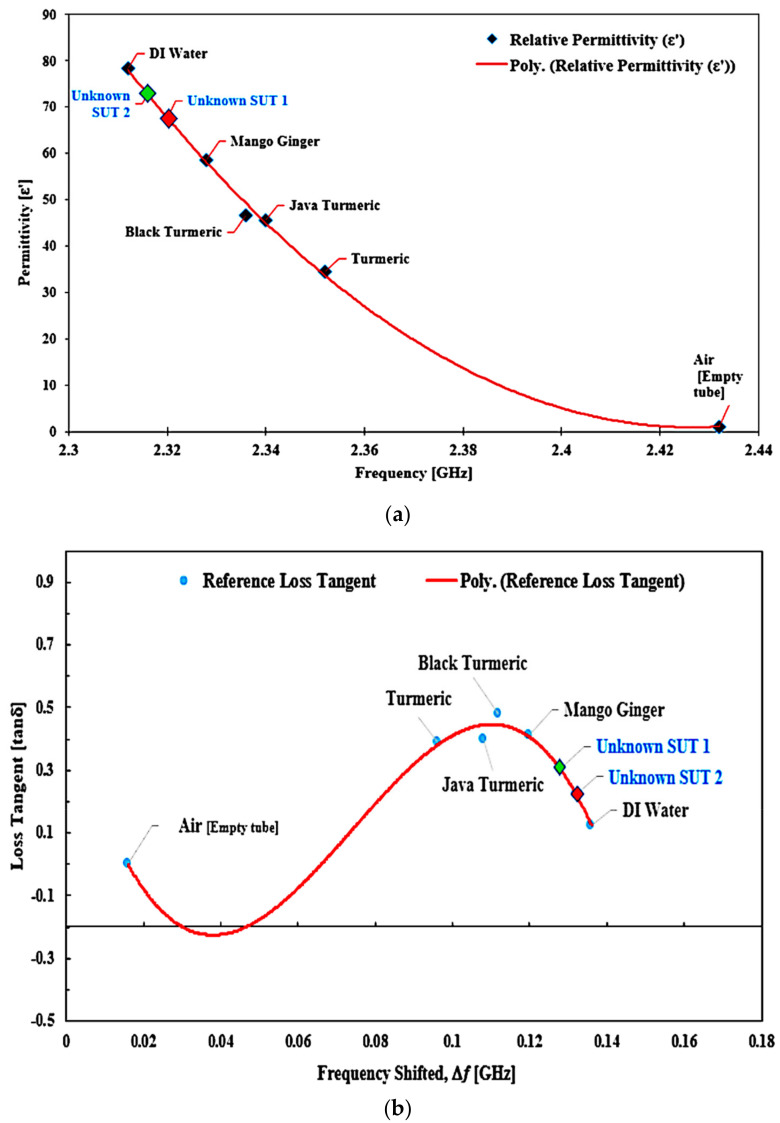
(**a**) Real permittivity and (**b**) loss tangent of the unknown semisolid SUTs compared to those of the known SUTs based on frequency.

To measure frequency, two types of unidentified semisolid samples were tested as described in Figure 25. The frequency variable was inserted in Equation (5) to evaluate the real permittivity value of the unknown SUTs. By using the same technique, the unknown value of loss tangent was determined using Equation (6).

**Figure 26:**
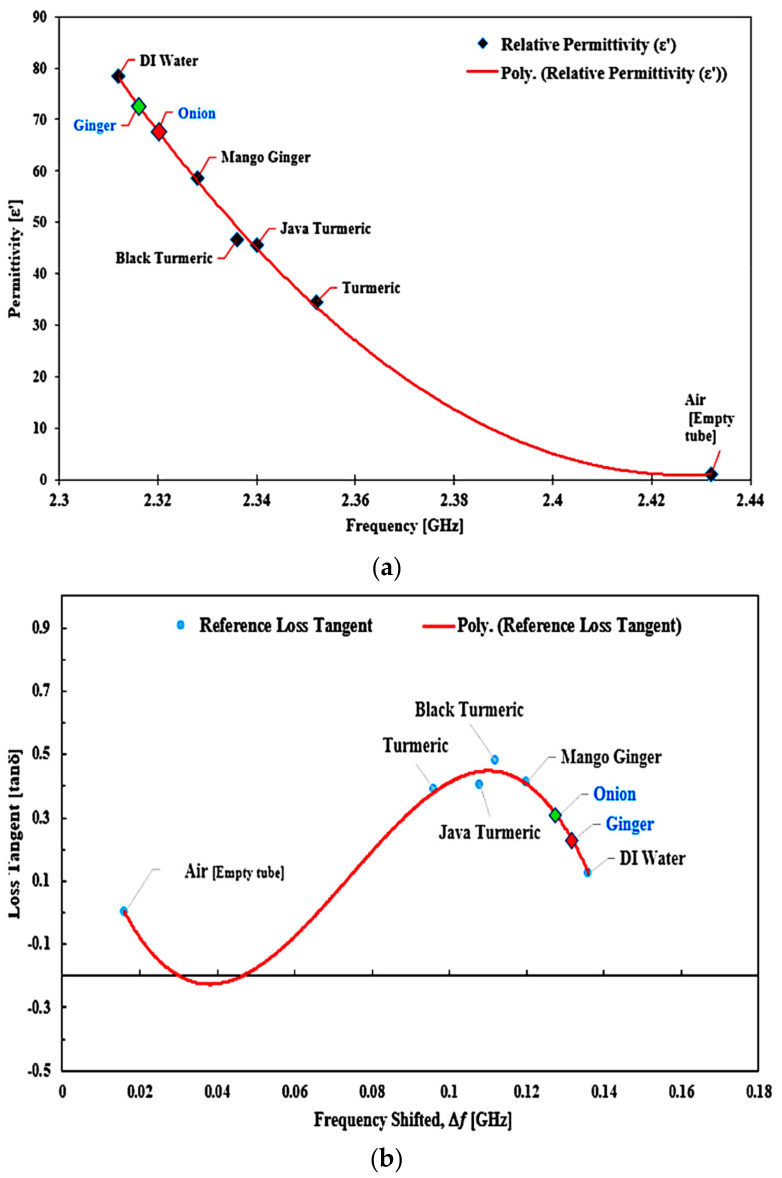
Position values of the unknown semisolid SUTs based on frequency and the calculated (**a**) real permittivity and (**b**) loss tangent.

After calculation, the unknown samples of onion and ginger were described as being relatively similar to those used by Karimi [64] and Racoti et al. [65], respectively. The experimental results of the real permittivity and loss tangent calculated using polynomial equations for each SUT are compared in Table 11 and displayed in Figure 26.

### 5.3. Sensitivity

The response of the resonant frequency relies on the dielectric constant of the materials. When a SUT is mounted on the maximum electric fields of the MDGS CSIW sensor, the electrical field of the resonator is disrupted. It was found that the resonant frequency shifts. In order to assess sensor performance, fractional variations in the resonant frequency were calculated for efficient permittivity described as sensitivity (*S*). Due to the relative shifts in frequency of the MDGS CSIW sensor, this led to relative changes in permittivity in the test samples, where an empty sample (air) was often used as a reference. The results illustrated in Table 12 show sensitivity of the various semisolid SUTs. Sensitivity can be calculated using Equation (7) below [64]:

*S* = Δ*f*/Δ*ε*′ (7)

Here, Δ*f* is the proportional disparity between the unloaded and loaded SUTs, Δ*f* = (*f_o_* − *f*_s_)/*f*_s_. At the same time, the interpretation of the dielectric constant Δ*ε* is described by air and the SUT’s dielectric constant Δ*ε*′ = (*ε*′ − (*ε*′)). To evaluate the performance of the sensor, fractional differences in the resonant frequency for an efficient dielectric constant were measured and plotted as sensitivity.

The maximum sensitivity of the MDGS CSIW sensor with a semisolid material is calculated as *S* = 2.864 MHz/*ε_r_* with permittivity variance. The sensitivity of the sensor is higher than in references ##1–17 in Table 13 because it has a larger E-field. Once the permittivity of the SUT is changed, the interaction of the electric field of the MDGS CSIW sensor eventually affects the resonant frequency shift. It should be noted that in the resonant perturbation technique, any change in the dielectric properties of the sample affects the resonant frequency shift and the sensitivity of the sensor. A comparison shows competitive performance of the proposed sensor configurations in terms of size, Q-factor, SUTs, techniques used, and sensitivity, as listed in Table 13.

## 6. Conclusions

In this work, we developed a high-Q-factor sensitivity microwave sensor based on the circular SIW approach operating at 2.45 GHz to characterize semisolid materials. The proposed circular SIW sensor was integrated with a mill-shaped DGS for a better sensitivity-matching circuit. We also investigated the sensor at different volumes of LUTs inside the PP tube and the optimal volume length was found to be 3.845 mm, which corresponds to 0.11 μL of semisolid samples. SUTs are filled into tubular polypropylene channels (PP) and loaded into the center hole of the MDGS CSIW resonator. The E-fields near the resonator affect the interaction with the SUTs, resulting in a strong and harmonic electric field at resonance, and the measured transmission response varies significantly. Through detailed measurements, the presented MDGS CSIW sensor can detect some standard semisolid samples and concentrations of SUT mixtures. Rogers RT/duroid 5880 was chosen as the substrate because it has a low electrical loss and a stable dielectric constant over frequency. A high-frequency structure simulator (HFSS) version 15.0 was used to simulate the proposed design of the MDGS CSIW sensor. The proposed MDGS CSIW sensor featured the best performance with high accuracy and the lowest average error detection of the real part permittivity of 1.86% compared to a commercial Agilent 85070E dielectric probe which has an error of 18.2%. The sensitivity of the proposed resonator proved to be high compared to other documented sensors and is high enough to easily characterize four different materials with different dielectric constants. The proposed MDGS CSIW sensor has many features such as low profile, ease of fabrication, high Q-factor and high sensitivity of about 700 and 2.864, making it a good candidate for semisolid material detection.

## Figures and Tables

**Table 1:** Specifications of the conventional CSIW design.

Design Specifications	
Material	RT/duroid 5880
Dielectric constant	2.2
Loss tangent	0.0009
Thickness of the substrate, h	3.175 mm
Thickness of copper, t	0.035 mm

**Table 2:** The values of the transverse mode, Bessel function [59].

*ρ_nm_*	*m* = 0	*m* = 1.0	*m* = 2.0	*m* = 3.0	*m* = 4.0
n = 1	2.4049	3.8318	5.1357	6.3802	7.5884
n = 3	5.5201	7.0156	8.4173	9.7610	11.0647
n = 3	8.6537	10.1735	11.6199	13.0152	14.3726

**Table 3:** Resonant frequencies for several TM modes of the CSIW.

Modes	Bessel Function	Frequency (GHz)					
		First Mode	Second Mode	Third Mode	Fourth Mode	Fifth Mode	Sixth Mode
TM_010_	2.4049	2.45					
TM_110_	3.8318	2.45	3.896				
TM_210_	5.1357	2.45	3.896	5.22			
TM_020_	5.5201	2.45	3.896	5.22	5.612		
TM_310_	6.3802	2.45	3.896	5.22	5.612	6.487	
TM_120_	7.0156	2.45	3.896	5.22	5.612	6.487	7.133

**Table 4:** Simulation performance of several diameters of the MDGS.

Diameter (D_MDGS_) in mm	Frequency (GHz)	Insertion Loss (dB)	E-Fields (v/m)
7.1	2.47	−2.69112	3.0041 × 10^4^
7.6	2.46	−4.52531	3.0263 × 10^4^
8.1	2.45	−3.66046	3.0673 × 10^4^
8.6	2.43	−3.64054	3.1411 × 10^4^
9.1	2.40	−3.94756	3.2743 × 10^4^

**Table 5:** Simulation datasets of the MDGS CSIW approach with the various semisolid SUTs.

SUTs	Frequency (GHz)	S21 (dB)	Frequency Changed (MHz)
**Without a tube**	2.45	−3.5610	0
**Air (empty tube)**	2.44	−3.545	10
**Turmeric**	2.356	−6.161	94
**Javanese turmeric**	2.343	−6.160	107
**Black turmeric**	2.338	−6.2495	112
**Mango ginger**	2.332	−6.162	118
**DI water**	2.316	−5.158	134

**Table 6:** Resonant frequency, Q-factor, and S21 magnitude (dB) simulated and measured for the MDGS CSIW sensor.

SUTs	Q-Factor	Simulation		Measurement	
		Frequency (GHz)	S_21_ (dB)	Frequency (GHz)	S_21_ (dB)
Without a tube	700	2.45	−3.561	2.448	−3.375
With a tube	300	2.44	−3.545	2.432	−2.392

**Table 7:** Repeated measurements of the dielectric value of the SUTs.

SUTs	Frequency, *f* (GHz)				Permittivity (*ε*′)			
	$\tilde{x}$	*f*_1_	*f*_2_	*f*_3_	$\tilde{x}$	*ε*′_1_	*ε*′_2_	*ε*′_3_
**Air (empty tube)**	2.432	2.4318	2.432	2.4322	1.001	0.9890	1.0014	1.0143
**Turmeric**	2.352	2.3517	2.3527	2.3521	35.554	33.8177	32.9428	33.4663
**Javanese turmeric**	2.34	2.3405	2.3409	2.3398	44.928	44.4198	44.0158	45.1314
**Black turmeric**	2.336	2.3365	2.3357	2.3361	49.095	48.5637	49.4151	48.9885
**Mango ginger**	2.328	2.3275	2.3279	2.3283	57.994	58.5754	58.1103	57.6469
**Dielectric water**	2.312	2.3118	2.3128	2.3114	78.050	78.3202	76.9761	78.8611

**Table 8:** Frequency shift with a different dielectric constant of semisolid SUTs.

SUTs	Relative Permittivity (*ε_r_*)	Simulation		Measurement	
		Frequency (GHz)	S_21_ (dB)	Frequency (GHz)	S_21_ (dB)
**Air (empty tube)**	1.0006	2.44	−3.545	2.432	−3.392
**Turmeric**	34.52	2.356	−6.161	2.352	−9.456
**Javanese turmeric**	45.6	2.343	−6.16	2.34	−10.205
**Black turmeric**	46.68	2.338	−6.2495	2.336	−9.686
**Mango ginger**	58.61	2.332	−6.162	2.328	−10.503
**Dielectric water**	78.4	2.316	−5.158	2.312	−7.385

**Table 9:** Comparison of the real permittivity and percentage error characterization between the proposed sensor and a commercial sensor for the various semisolid SUTs.

SUTs	Frequency Shifting (GHz)	Reference Relative Permittivity [56]	Proposed Sensor		* Commercial Sensor	
			Relative Permittivity (*ε′*)	Error (%)	Relative Permittivity (*ε*′)	Error (%)
**Air (empty tube)**	2.432	1.0006	1.006	10.03	1.0089	0.288
**Turmeric**	2.352	34.52	34.317	0.59	52.29	55.838
**Javanese turmeric**	2.34	45.6	45.047	1.21	54.45	21.194
**Black turmeric**	2.336	46.68	46.285	0.85	48.88	0.438
**Mango ginger**	2.328	58.61	59.366	1.29	41.14	29.062
**Dielectric water**	2.312	78.4	78.177	0.28	79.92	2.396
**Average error**			**1.86%**		**18.2%**	

* Agilent 85070E dielectric probe.

**Table 10:** Comparison of the percentage error of loss tangent of the SUTs (semisolid) between the proposed sensor and the commercial sensor.

SUTs	Frequency Shifting (Δ*f*)	Reference Ideal Loss Tangent	Proposed Sensor		* Commercial Sensor	
			Loss Tangent (*tan δ*)	Error (%)	Loss Tangent (*tan δ*)	Error (%)
Air (empty tube)	0.016	0	0.0001	**0**	0.0001	0
Turmeric	0.096	0.39	0.3798	2.6	0.253	35.135
Javanese turmeric	0.108	0.4	0.4456	11.4	0.252	36.905
Black turmeric	0.112	0.48	0.4459	7.1	0.261	45.615
Mango ginger	0.12	0.41	0.4061	1.0	3.83	6.702
Dielectric water	0.136	0.123	0.1253	1.9	0.197	45.43
**Average error**			**4%**		**28.3%**	

* Agilent 85070E dielectric probe.

**Table 11:** Calculated complex permittivity of several known and unknown semisolid SUTs.

SUTs	*f* (GHz)	Δ*f* (GHz)	Reference			Calculated		
			*ε*′	*tan δ*	*ε*″	*ε*′	*tan δ*	*ε*″
Air (empty tube)	2.432	0.016	1.0006	0	0	1001	0.0001	0.0001
Turmeric	2.352	0.096	34.52	0.39	13.46	33.554	0.3798	12.7438
Javanese turmeric	2.340	0.108	45.6	0.4	18.24	44.928	0.4456	20.0199
Black turmeric	2.336	0.112	46.68	0.48	22.41	49.095	0.4459	21.8915
Mango ginger	2.328	0.12	58.61	0.41	24.03	57.994	0.4061	23.5514
Onion	2.320	0.128	64	0.218	14	67.646	0.3023	20.4494
Ginger	2.316	0.132	71.42	0.199	14.23	72.754	0.224	16.2969
Dielectric water	2.312	0.136	78.4	0.123	9.64	78.050	0.1253	9.7797

Note: *f*: frequency, Δ*f:* frequency shifting *ε*′: real permittivity, *tan δ*: loss tangent, *ε*″: imaginary permittivity.

**Table 12:** Sensitivity of the various semisolid SUTs.

SUTs	Frequency (GHz)	Δ*f* (MHz)	*ε_r_*	Δ*ε_r_*	*S* (MHz/*ε_r_*)
Air (empty tube)	2.432	16	1.0006	0	0
Turmeric	2.352	96	34.52	33.519	2.864
Javanese turmeric	2.340	108	45.6	44.599	2.422
Black turmeric	2.336	112	46.68	45.679	2.452
Mango ginger	2.328	120	58.61	57.609	2.083
Dielectric water	2.312	136	78.4	77.399	1.757

Note: Δ*f*: frequency shifted, *εr*: relative permittivity, Δ*εr*: variants of relative permittivity, *S*: sensitivity.

**Table 13:** Comparison with the existing studies on sensors.

#	Reference	Sensor Size (mm)	Used Techniques	SUTs	Frequency Band (GHz)	Q-Factor	Sensitivity (*S*)
1	[25]	80 × 40 × 0.8	Metamaterial coupling	Ethanol and methanol	2.5	Not reported	0.27
2	[28]	80 × 25 × 0.8	Loss-compensated SRR	Glucose	1.156	190	Not reported
3	[29]	26 × 30 × 26.5	Waveguide with a loop slot	Ethanol and dielectric water	91	Not reported	Not reported
4	[30]	112.96 × 49.16 × 3.175	Multiple split-ring resonator	Ethanol, methanol, and air	2.1	525	Not reported
5	[33]	75 × 33 × 1	SIW	Ethanol and water	5.85	334.6	Not reported
6	[34]	50 × 40 × 1.6	EMSIW	Ethanol, methanol, and dielectric water	4.6	Not reported	1.5
7	[35]	45 × 45	SIW	Semisolid and non-solid materials	2.5	152	Not reported
8	[36]	Not reported	SICR	Microfluidic applications	92.65	Not reported	0.061
9	[66]	25 x 35 x 1.6	SRR	Microfluidic sensor applications	4–6	230	Not reported
10	[67]	25 × 30 × 1.54	CCSR	Ethanol, methanol, and milk	2.4	Not reported	Not reported
11	[68]	30 × 25 × 1.6	CSSRRs	AIR, HDPE, and PVC	5.35 and 7.99	267.5	0.04
12	[69]	46 × 46 × 1.6	OCSRRs	Ethanol, methanol, and dielectric water	0.9	Not reported	4.3
13	[70]	28 × 20 × 0.75	CSRR	Ethanol and water	2.85 and 2.96	145	3.0
14	[71]	35 × 25 × 1.6	SRR	Ethanol, methanol, and dielectric water	2.45	31	0.214
15	[72]	40 × 20 × 1.6	OSRR	Ethanol, methanol, dielectric water	2.5–3.5	Not reported	Not reported
16	[73]	30 × 13 × 0.508	MML	Solid	5.65	217	3.25
17	[74]	38 × 35.4 × 15.73	GWCR	Ethanol, methanol, and air	5.96	66.8	0.156
**This work**		**69 × 69 × 3.175**	**MDGS-CSIW**	**Javanese turmeric, mango ginger, black turmeric, turmeric, and distilled water (DI)**	**2.45**	**700**	**2.864**
